# Optimization of protein extraction and digestion workflows for proteomic analysis of saliva, salivary stones and kidney stones

**DOI:** 10.3389/fmolb.2026.1769952

**Published:** 2026-03-16

**Authors:** Natalia Musiał, Martyna Iwaniec, Inez Mruk, Michał Puchalski, Dmitry Tretiakow, Andrzej Skorek, Konrad Szydłowski, Jan Szlęzak, Paulina Czaplewska

**Affiliations:** 1 Laboratory of Mass Spectrometry, Intercollegiate Faculty of Biotechnology, University of Gdansk, Gdansk, Poland; 2 Laboratory of Biopolymers Structure, Intercollegiate Faculty of Biotechnology, University of Gdansk, Gdansk, Poland; 3 Clinical Department of Otolaryngology, Academy of Applied Medical and Social Sciences, The Nicolaus Copernicus Hospital in Gdansk, Copernicus Healthcare Entity, Gdansk, Poland

**Keywords:** biocalcification, biomarkers, kidney stones, proteomics, saliva, salivary stones, sample preparation, sonication

## Abstract

**Background:**

Salivary and kidney stone diseases are associated with pathological calcification, yet their molecular composition remains incompletely characterized. Proteomic analysis of saliva and stone material may provide valuable insights into these processes; however, such analyses are technically challenging and strongly dependent on efficient and reproducible sample preparation workflows.

**Methods:**

In this study, we systematically evaluated and optimised protein extraction and digestion conditions for proteomic analysis of saliva, salivary stones and kidney stones. Different lysis buffers, sample amounts, sonication parameters and digestion strategies were tested. Additional biological materials, including salivary gland tissue and *Raoultella ornithinolytica*, were analysed to assess the broader applicability of the developed workflows.

**Results:**

Optimised workflows significantly improved peptide and protein identification, reduced the proportion of missed cleavages and enhanced sequence coverage across multiple biological matrices. Sonication-assisted protocols proved particularly effective for stone-derived materials, although optimal conditions varied depending on the sample type.

**Conclusion:**

This pilot study demonstrates that careful optimisation of protein extraction and digestion is essential for reliable proteomic analysis of challenging biomaterials such as saliva and pathological calcifications. The workflows established here provide a robust methodological foundation for future large-scale and biomarker-oriented proteomic studies of stone disease and related calcification disorders.

## 1 Introduction

Sialolithiasis is a relatively rare and underrecognised condition characterised by the formation of calcified deposits known as salivary stones, or sialoliths, affecting approximately 1%–2% of the population. These stones form within the salivary ducts or glands. While most cases involve the submandibular glands, the parotid and sublingual glands are affected less commonly. Clinical problems arise when a sialolith grows to a size that blocks the salivary duct, disrupting the flow of saliva. This situation may result in severe pain, especially during meals, swelling of the affected salivary gland area, and, in some cases, fever. The condition is frequently accompanied by bacterial infection and purulent inflammation of the salivary gland tissues. Currently, the only available treatment for this condition is surgical removal of the stones (Sigismund et al., 2015; Kraaij et al., 2014; Kopeć et al., 2013; Huoh and Eisele, 2011; Delli et al., 2014).

From a clinical perspective, salivary and kidney stone diseases are associated with recurrent symptoms, repeated diagnostic procedures and, in many cases, surgical interventions, which collectively impose a substantial burden on healthcare systems. The lack of reliable molecular markers limits early diagnosis, risk stratification and monitoring of disease progression, resulting in predominantly reactive rather than preventive clinical management.

Integration of optimised proteomic workflows into translational research pipelines may, in the long term, support the development of more precise diagnostic and prognostic tools. Such advances could reduce the frequency of invasive procedures, improve patient stratification and enable earlier intervention, thereby lowering treatment costs and improving quality of life. In the short term, standardised and reproducible sample preparation protocols are essential to ensure data comparability across clinical centres, which is a prerequisite for the implementation of proteomics-based approaches in routine clinical and laboratory practice.

According to the proposed classification and results of the spectroscopic studies (Tretiakow et al., 2021), sialoliths can be categorised into three types: calcified, lipid, and mixed. This classification suggests that CAL and LIP stones have different developmental pathways, while MIX stones initially form as CAL stones but switch to a LIP pattern at some stage. The balance of calcium and lipids is disturbed during these developmental phases.

The key unresolved issue remains the mechanism of biocalcification that leads to the formation of sialoliths. The structure of formed sialoliths is varied and complicated, often consisting of an inner core surrounded by thin outer layers. Depending on the type of sialolith, these layers can be inorganic, with a well-understood structure, or organic, with an incompletely described composition. The organic layers contain proteins, lipids, and bacteria, including both commensal oral flora and pathogenic microorganisms. According to one theory of sialolith formation, bacteria may influence the biocalcification process, contributing to stone formation (Sabot et al., 2012; Szalma et al., 2013; Stelmach et al., 2016; Tretiakow et al., 2020; De Grandi et al., 2019; Fusconi et al., 2016; Parsek and Singh, 2003; Perez-Tanoira et al., 2019; Teymoortash et al., 2009). Conversely, previous research indicates that epithelial cells of the salivary gland can secrete peptides and proteins involved in digestion, lubrication, mineralisation, tissue coating, buffering, and antimicrobial activity (Sabbadini and Berczi, 1998; Amado et al., 2014; Castagnola et al., 2012).

Based on our working hypothesis, the level of proteins secreted by salivary glands, or those resulting from bacterial infection, can affect the balance of calcium and lipids, thereby leading to the formation of sialoliths. Identification of molecular features associated with biocalcification may, in the long term, contribute to improved understanding of stone formation processes in sialolithiasis. The results from our last two papers have allowed us to identify some potential biomarkers, even considering the classification of salivary stones (Czaplewska et al., 2021; Musiał et al., 2023). However, the translational applicability of these and other published proteomic studies remains limited by differences in sample preparation strategies, analytical workflows and data reproducibility, underscoring the need for methodological standardisation prior to clinical implementation. To confirm these potential markers with higher confidence, a comparison of the proteome of sialoliths and saliva from patients with sialolithiasis with that of the proteome of saliva from healthy donors should be conducted. Potential biomarkers for sialolithiasis are probably present in both salivary stones and saliva, despite saliva being a biological material that can be unstable and sensitive to various conditions. Even if the opposite proves true, such comparisons can still yield clinically valuable conclusions. A critical comparison between saliva-derived proteomes from patients with salivary stones and healthy donors represents a necessary step toward future studies aimed at identifying disease-associated molecular patterns. Mass spectrometry coupled with liquid chromatography enables high-throughput identification and quantification of numerous proteins in well-prepared samples and represents a key technology in translational biomedical research. However, satisfactory results depend on the development and optimisation of protein sample preparation and MS acquisition methods. This optimisation is crucial for obtaining accurate and reproducible proteomic analysis results, especially given the risk of compromising the quality of clinical material work due to the limited number of available samples. Therefore, developing optimised and reproducible sample preparation workflows is imperative to ensure data comparability, scalability and future integration of proteomics-based approaches into clinical and laboratory practice. Since few protocols are described for stone samples, it would be advantageous to develop a method suitable for different stone types, such as kidney stones or dental calculus, which would enable analogous experiments in other studies. To determine if a universal protocol for any biological material is feasible, three additional types of biological material were tested alongside saliva and salivary stones: kidney stone samples, salivary gland samples, and a culture of *Raoultella ornithinolytica*. In addition to saliva and stone-derived materials, a bacterial culture of *R. ornithinolytica* was included in this study. This microorganism was not selected as a representative of oral microbiota; instead, it served as an additional and biologically distinct matrix to evaluate the versatility and robustness of the optimised protein extraction and digestion workflows. Bacterial cells differ substantially from human tissues and calcified materials in terms of cell structure and protein composition, thereby providing a stringent test for the applicability of the developed protocols across diverse biological samples.

Importantly, recent studies have reported associations between *R. ornithinolytica* infections and urinary stone formation, particularly in pediatric patients, highlighting its potential relevance in the context of pathological calcification (Karacı and Yaşar, 2020; Pi et al., 2020). Therefore, inclusion of this microorganism also allowed us to explore the performance of the optimised workflows in a bacterial model linked to urinary stone disease (Hajjar et al., 2020).

Although mass spectrometry-based proteomics has become a powerful tool in biomedical research, its translation into routine clinical diagnostics remains limited, largely due to variability in sample preparation and insufficient standardisation of analytical workflows. This challenge is particularly pronounced for unconventional and complex biological materials, such as calcified tissues, biological stones or saliva, which are rarely addressed in standard clinical proteomics pipelines.

Recent studies have demonstrated the feasibility of applying proteomic approaches to stone disease and related calcification disorders; however, methodological heterogeneity across studies hampers direct comparison and clinical translation. Therefore, the development and validation of robust, reproducible and adaptable sample preparation workflows represents a critical step towards the biotechnological integration of proteomics into clinical research and, ultimately, routine diagnostic frameworks.

To enhance the efficiency of the lysis and digestion process, a sonicator, a standard laboratory equipment for protein extraction and digestion, was employed. The primary goal was to reduce the time required for the entire sample preparation process while maintaining high efficiency (Carvalho et al., 2020). To validate the use of the sonicator, samples were also processed with a barocycler. Pressure cycling technology is another well-established method for improving standard protocols and has been validated in combination with mass spectrometry experiments for both data-dependent and data-independent acquisition.

Finally, to confirm the presence of a common group of proteins in both saliva and salivary stones, a comparative analysis of detected proteins was performed. It suggests that a quantitative analysis for both sample types would be reasonable to identify potential biomarkers of sialolithiasis. Furthermore, comparing proteins extracted from salivary and kidney stones can reveal potential overlaps, highlighting possible universal proteins involved in pathological calcification. This approach could indicate the possibility of detecting crucial and universal proteins involved in the biocalcification process across various diseases.

## 2 Materials and methods

This study was designed as a methodological optimisation and proof-of-concept investigation. Due to the limited availability of clinical material, optimisation experiments were performed on pooled samples prepared separately for each biological material type. Each tested condition was analysed in three technical replicates. No formal power analysis was conducted, as the primary objective of the study was methodological optimisation rather than hypothesis-driven statistical inference.

### 2.1 Collecting samples

Salivary stone samples were collected from patients under the care of the Department of Otolaryngology at the Medical University of Gdańsk. Patients were included in the study only after signing the necessary written consent and approval by the Independent Bioethics Commission at the Medical University of Gdańsk. The process of collecting salivary stone samples was the same as described earlier (Musiał et al., 2023), optimised and standardised according to the applicable routine protocol from the Clinic of Otolaryngology, Department of Oral and Maxillofacial Surgery, at the University Clinical Centre in Gdańsk. Sialoliths were removed during endoscopic, transoral or transcervical surgery. After that, the salivary stone samples were washed with the use buffer (25 mM NH_4_HCO_3_) and then stored in sterile Falcon tubes at 80 °C for further experiments. Twenty salivary stones of submandibular origin were collected for the study.

Saliva samples were collected from fifteen healthy volunteers using the Salivette® collection system (Sarstedt, Germany) according to the manufacturer’s protocol. Participants were instructed to refrain from eating, drinking and oral hygiene procedures for at least 1 h prior to sample collection. Immediately before sampling, participants rinsed their oral cavity with water for 1 min. After collection, saliva was recovered by centrifugation (20 min, 15,000 g, 4 °C) of the Salivette devices, supplemented with a protease inhibitor cocktail to prevent proteolytic degradation, and stored at −80 °C until further analysis. All kidney stone samples were collected from patients treated in the Department of Urology at the Medical University of Gdańsk. All patients were included in the study only after signing the necessary written consent and approval by the Independent Bioethics Commission at the Medical University of Gdańsk. The process of collecting kidney stone samples was the same as described earlier (Musiał et al., 2023), optimised and standardised according to the applicable routine protocol from the Department of Urology at the Medical University of Gdańsk. Kidney stones were removed during endoscopic or open surgery. After that, the kidney stone samples were washed with the use buffer (25 mM NH_4_HCO_3_) and then stored in sterile Falcon tubes at 80 °C for further experiments. As part of the research, 10 kidney stones were collected.

Salivary gland samples (16 parotid samples) were collected in the same way as described earlier (Puchalski et al., 2024). A fragment of salivary gland tissue (approximately 25 mg per experiment) was collected after parotidectomy and then placed in a freezer at a temperature of −20 °C within 4 h of collection and processed later.

*Raoultella ornithinolytica* MF1 culture was purchased from the Laboratory of Biologically Active Compounds at the Intercollegiate Faculty of Biotechnology of the University of Gdańsk and the Medical University of Gdańsk. 20mL of *R. ornithinolytica* MF1 culture was growing at 28 °C in tryptone soya broth (TSA, Oxoid) for 16 h with shaking (150 rpm).

### 2.2 Protein extraction

To perform protein extraction and to maintain homogeneity of the material, whole salivary stones and kidney stoneswere crushed into powder using a mortar. All the obtained material from 20 salivary stones (exactly the same examined and described earlier (Musiał et al., 2023)) was mixed and pooled to obtain a homogeneous sample, from which defined amounts of 25 mg or 50 mg were used for subsequent optimisation steps.

Salivary gland tissue samples were fully homogenised using a hand-held mechanical homogeniser (OMNI International TH115) until a visually uniform tissue suspension was obtained. For protein extraction, aliquots corresponding to 100 mg of homogenised tissue were used for each lysis experiment. Homogenisation of the entire tissue material ensured a consistent sample composition across all optimisation steps. A fixed amount (100 mg) of each type of sample was treated with 250 µL of lysis buffer with SDS (1% or 3% SDS, 100 mM Tris–HCl, pH 8.0, 50 mM DTT) or RIPA buffer (Thermo Fisher Scientific). Control samples were treated in a standard way–after adding the lysis buffer, they were incubated at 95 °C with mixing (lysis buffers with SDS and RIPA buffer with heating) or on ice with occasional mixing (RIPA buffer) for 15 min. Samples were centrifuged at 14,000 × g for 15 min at 4 °C, and the supernatant was collected. Protein extraction enhanced by sonication was performed using 15-minsonication at 20 °C with varying amplitudes and cycle durations as described in Table 1. The following two stages focused on sonicator-aided lysis: the duration of cycles and amplitude of sonication were optimised. Regarding the duration of cycles, four different conditions were applied: 5 s ON/5 s OFF, 15 s ON/5 s OFF, 30 s ON/5 s OFF, and 30 s ON/15 s OFF. In the case of sonication amplitude, four different values were tested: 25%, 50%, 75%, and 100%. During these two stages, samples were also processed without sonication (control).

**TABLE 1:** Results of optimisation process, where the most optimal conditions of protein extraction are presented for each step and each type of biological material: saliva, salivary stones, kidney stones, salivary glands and *Raoultella ornithinolytica*. These variants were chosen, and the next stages of optimisation were followed accordingly.

Biological material Condition	Saliva	Salivary stones	Kidney stones	Salivary glands	*Raoultella ornithinolytica*
Lysis buffer	RIPA HOT	SDS 3%	SDS 3%	SDS 1%	SDS 1%
Amount of material	50 µL	50 mg	50 mg	50 µL	50 µL
Duration of cycles	15s ON/5s OFF	15s ON/5s OFF	30s ON/15s OFF	5s ON/5s OFF	30s ON/15s OFF
Amplitude of sonication	50%	25%	75%	25%	50%
FASP digestion	Standard overnight and 15-min sonicatior-aided	Standard overnight and 15-min sonicatior-aided	15-min sonicatior-aided and 2 × 15-min sonicatior-aided + standard overnight	15-min sonicatior-aided and 2 × 15-min sonicatior-aided + standard overnight	Standard overnight and 15-min sonicatior-aided

Then, there was incubation at 95 °C with mixing or incubation on ice with occasional mixing for 15 min. After that, sonication under the same conditions as earlier was repeated, and the samples were centrifuged (15 min, 15,000 g, 4 °C) to collect the supernatant.

Barocycler-aided protein extraction was performed based on a previously published protocol (Pirog et al., 2021), with minor modifications. 25 mg (salivary stones and kidney stones) or 25 µL of sample was treated with 125 µL of lysis buffer. The entire combination for each sample type was transferred into PCT microtubes (Pressure Biosciences, MA, United States) and sealed with a microcap. The process of lysis was performed with 90 pressure cycles (25 s at 45 kpsi and 10 s at low pressure) at 80 °C in a Barocycler 2320EXT. After this processing, samples were centrifuged (15 min, 15,000 g, 4 °C), and the supernatant was collected.

### 2.3 Digestion of extracted proteins

Digestion protocols were based on previously described standard FASP and sonicator-aided FASP approaches (Carvalho et al., 2020; Wiśniewski et al., 2009a) The first used protocol was standard FASP, where samples were digested overnight with trypsin at 37 °C on a 10 kDa molecular weight cutoff membrane. Samples digested according to the second protocol were processed with trypsin during sonication for only 15 min (the optimal duration and amplitude values for each type of sample) at 37 °C on a 10 kDa molecular weight cutoff membrane. The last tested protocol combined overnight digestion with sonication: first, samples were sonicated for 15 min at 37 °C on a 10 kDa membrane; then, they were incubated overnight at 37 °C; and finally, they were sonicated for an additional 15 min. Ultimately, the obtained peptide fractions were prepared for MS analysis by final clean-up on C18 (exchange disks, 3M Empore™) StageTips, as described in the protocol (Rappsilber et al., 2007).

### 2.4 Qualitative (DDA) LC-MS/MS analysis

This part was performed as described earlier (Musiał et al., 2023). LC–MS/MS analysis was performed on a Triple-TOF 5600+ mass spectrometer (AB Sciex LLC, Framingham, MA, United States) connected to an Ekspert MicroLC 200 Plus System (Eksigent, Dublin, CA, United States). The Analyst TF 1.7.1 software (SCIEX) controlled the whole system. The chromatographic gradient for each MS run was 11%–42% B (A: H_2_O + 0.1% FA; B: 100% can + 0.1% FA) in 60 min. A ChromXP C18CL column (3 µm, 120 Å, 150 × 0.3 mm) was used to perform the chromatographic separation. The spectra were recorded in information-dependent acquisition (IDA) mode to facilitate qualitative analysis and library construction. Each cycle comprised precursor spectra accumulation in 100 ms in the range of 400–1,200 m/z, followed by the accumulation of the top 20 precursor ion spectra in 50 ms in the range of 100–1,800 m/z, resulting in a total cycle time of 1.15 s. Formerly fragmented precursor ions were dynamically excluded.

The results of MS analysis performed in IDA mode were analysed in PEAKS Studio 11.5 software with the following settings: instrument—TripleTOF, fragmentation method—CID, acquisition—DDA, parent mass error tolerance - 15.0 ppm, fragment mass error Tolerance - 0.05 Da, precursor mass search type - monoisotopic, digestion—trypsin, reduction and alkylation of proteins (fixed modification—carbamidomethylation; variable modification—formylation and oxidation, maximum allowed variable PTM per peptide — 2, proteins unique peptides ≥2, FDR (Protein Group) - 1.0%. The data were analysed against the *Homo sapiens* database (UniProt, 19 November 2023) and the *R. ornithinolytica* (formerly *Klebsiella ornithinolytica*) database (UniProt, 19 October 2023). The mass spectrometry proteomics data have been deposited to the ProteomeXchange Consortium *via* the PRIDE (Perez-Riverol et al., 2025) partner repository with the dataset identifier PXD069065.

### 2.5 Statistical analysis and visualisation

The obtained data were processed using the Excel package (Microsoft). For data visualisation in Excel, the SRplot tool (Tang et al., 2023), Cytoscape 3.9.1 (Shannon et al., 2003) and Python with the matplotlib package (Hunter, 2007) were used. The enrichment analysis was performed, utilising STRING 12.0 (Szklarczyk et al., 2023) for this purpose.

## 3 Results

The results presented in this section summarise the stepwise optimisation of protein extraction and digestion workflows for proteomic analysis of challenging biological materials, with particular emphasis on saliva and pathological calcifications. Given the distinct physicochemical properties of these sample types, optimisation outcomes were evaluated across multiple biological matrices, including salivary stones, saliva, kidney stones, salivary gland tissue and bacterial cells, to assess the robustness and adaptability of the developed approach.

Optimisation was performed in a sequential manner, with key parameters selected at each stage based on their impact on proteomic performance metrics, including protein and peptide identification, missed cleavage rates and sequence coverage. The resulting optimal conditions were subsequently applied in downstream analyses, and a summary of the selected parameters for each sample type is provided in Table 1. The following subsections describe the outcome of each optimisation step.

**FIGURE 1:**
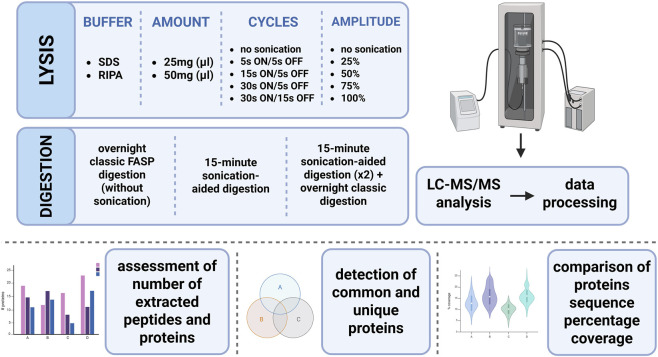
Graphical visualisation of study workflow (Created with BioRender).

After optimising the protocol for protein extraction, the next part of the optimisation process was digestion. In this step, the standard FASP digestion approach was used; however, to increase the number of identified proteins, this digestion method was modified by applying sonication. Two different methods were tested: the first protocol focused on maximising the number of detected proteins, so two 15-min sonication-aided treatments were applied during trypsin digestion, while classic overnight incubation was maintained. The second tested protocol was focused on reducing digestion time, so only 15-min sonication-aided digestion was applied. Standard FASP with overnight classic digestion was also tested (as a control). The workflow of process optimisation is presented in Figure 1.

An additional step involved comparing sonicator-aided lysis with barocycler-aided lysis. Sonication is not the only way of enhancing sample processing; that is why other commonly used method was tested to check the validity of using a sonicator.

Each step was assessed based on different values. The first criterion was the number of identified peptides and proteins for each condition–here, the highest possible value is desirable. The second criterion is the percentage value of missed cleavages, which indicates the proportion of identified peptides that were detected with missed cleavages; here, the lowest possible value is desirable. For different conditions, the overlap of identified proteins was examined to determine the number of common and unique proteins detected under each condition. The last criterion was the distribution of percentage values of coverage of identified proteins, comparing them to the proteins in the applied protein database. Results of proteomic analysis of tested samples during each step of optimisation are presented in Supplementary Material (Supplementary Materials S1–S5). A comprehensive summary of the optimal sonication duration and amplitude values selected for each biological material is provided in Table 1.

### 3.1 Optimisation of sample amount and choice of lysis buffer

**FIGURE 2:**
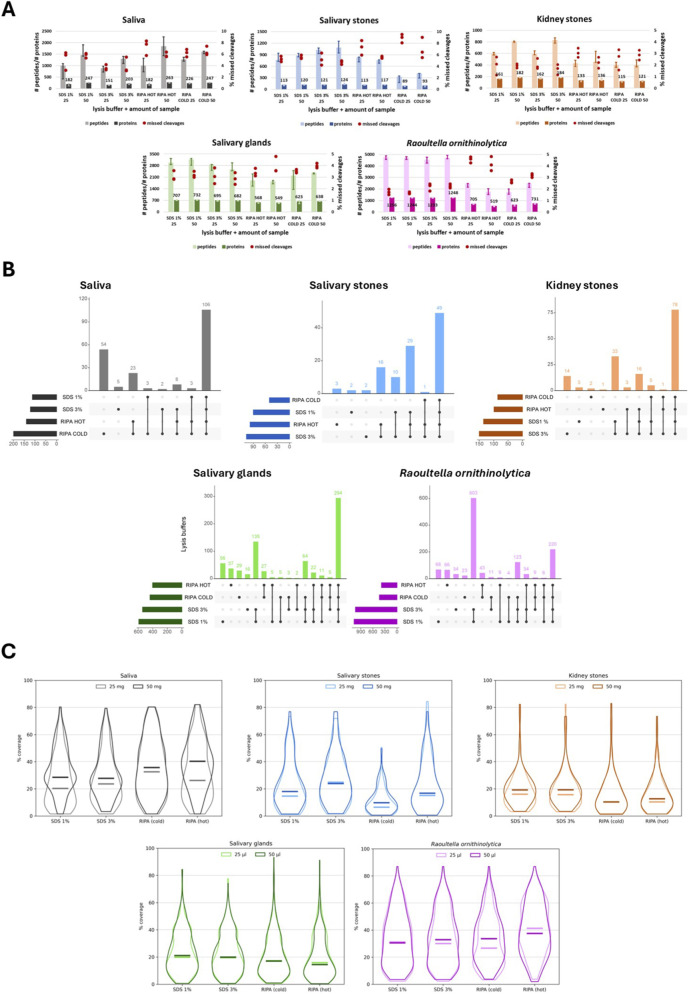
Presentation of proteomic data obtained for different lysis buffers and sample amounts, biological materials: saliva, salivary stones, kidney stones, salivary glands, and *Raoultella ornithinolytica*. **(A)** Bar charts show the median number of identified peptides and proteins for each applied sample amount and lysis buffer; red dots indicate the percentage of missed cleavages detected in three technical replicates. **(B)** Upset plots illustrate the overlap of identified proteins across different lysis buffers. **(C)** Violin plots present the distribution of sequence coverage percentages for identified proteins compared to the applied protein databases, with horizontal lines indicating media.

The first step of optimisation of sample amount and choice of lysis buffer was assessment of applied conditions based on the number (median) of identified peptides and proteins and percentage value of missed cleavages for three technical replicates (Figure 2A). RIPA buffer was selected as one of the lysis media due to its ability to solubilize a broad range of cellular proteins, including cytoplasmic, membrane and nuclear fractions, facilitated by the presence of ionic detergents such as SDS and deoxycholate, which disrupt protein–protein interactions and enhance extraction efficiency compared to milder buffers (Sube et al., 2019).

For comparative purposes, sonicator-assisted lysis was performed under both cold conditions (to preserve protein integrity) and hot conditions (especially in SDS-containing buffers, where heat can enhance denaturation and solubilization of complex protein assemblies).

SDS, a strong ionic detergent used in some of the tested lysis conditions, has been recommended in proteomic protocols at concentrations of ∼1% and above 3%–4% to improve protein solubilization, although care must be taken due to its potential interference with downstream MS analysis (Gan et al., 2021).

For saliva samples, the higher volume of samples (50 µL) allows for the identification of more proteins in the presence of all types of lysis buffers. Both the number of detected peptides and proteins is higher for the lysis buffer with 1% SDS compared to the 3% SDS buffer. In the case of the RIPA buffer, incubation at 95 °C has a positive impact on the number of identified peptides and proteins. Comparing the buffer with SDS and RIPA buffer applied to a higher volume of sample, the values are similar. For salivary stone samples, the worst results are obtained when samples are treated with RIPA buffer and incubated on ice. For the remaining variants, the values are quite similar to one another. However, the higher sample amount (50 mg) again facilitates the identification of more proteins. In the case of kidney stones, the amount of sample and the type of lysis buffer have a significant influence on the number of detected peptides and proteins–a higher amount of material (50 mg) and buffer with SDS allows for a higher quality of proteomic analysis. For salivary gland samples, there are no significant differences between lower and higher amounts of processed material; however, visible differences are observed between the various lysis buffers applied. Again, the buffer with SDS is more effective in detecting a wider range of peptides and proteins. There is a similar situation with the *R. ornithinolytica* samples–RIPA buffer is a less compelling option for identifying a high number of peptides and proteins. In general, higher volumes (50 µL) of treated samples yielded better results; however, for heated RIPA buffer, more peptides and proteins were identified in the case of lower volumes. The most significant difference visible across all tested types of biological material is the percentage value of detected missed cleavages–unfortunately, the values are significantly higher for RIPA buffer.

Based on upset plots, it is possible to verify the overlap of identified proteins, allowing for the distinction between common and unique proteins identified by applying a given variant of the protocol (Figure 2B). First, proteins detected in overlapping patterns across three replicates of each variant were selected. Then, proteins from two different sample amounts were chosen that overlapped. Then, these protein sets for the applied lysis buffers were compared with one another. It also indicates which variant may be the most repetitive by comparing the number of overlapping proteins within technical replicates. For saliva, 106 common proteins have been identified using all of the lysis buffer types. There are no significant differences between lysis buffers, except for RIPA buffer (with heating and incubation on ice), which detected 23 additional proteins.

Furthermore, performing lysis using RIPA buffer with incubation on ice enabled the identification of 54 additional proteins. This variant is also the most favourable, considering repeatability. 49 the same proteins were extracted from salivary stones using all the tested buffers. Using the RIPA buffer with ice incubation does not allow for the detection of almost any specific proteins. Buffers with SDS cause the extraction of 10 additional proteins. Besides, by applying RIPA buffer with heating and a buffer with a higher concentration of SDS (which also shows the highest repeatability), it is possible to detect 16 more proteins. Regarding kidney stones, 78 proteins remain the same regardless of the lysis buffer used. 33 specific proteins are extracted after lysis with a buffer containing SDS, and 14 more proteins after increasing the concentration of SDS from 1% to 3%. A similar situation exists for salivary glands–with buffers containing SDS, it is possible to identify 135 different proteins. However, a lower concentration of SDS has a more positive influence on the detection of unique proteins and repeatability. The most visible difference appears for *R. ornithinolytica* samples–there are only 220 common proteins for all the applied types of lysis buffer, but applying an SDS-based buffer allows the extraction of 603 more proteins. A buffer with a lower concentration of SDS allows for higher repeatability and the extraction of a greater number of unique proteins.

A different criterion for optimising the protocol is the distribution of percentage values of coverage of identified common proteins, compared to the applied protein database. There are overlapped violin plots for each variant of the applied lysis buffer, presenting the distribution of coverage values taking into account the amount of processed biological material (Figure 2C). For saliva samples, the higher volume (50 µL) generally increases the coverage of identified proteins. The lowest values are associated with the use of SDS-based buffers, and the highest values are obtained for samples treated with heated RIPA buffer. The same buffer, but combined with incubation on ice, has a significantly negative influence on the coverage of proteins extracted from salivary stones. Heating of this buffer allows for obtaining similar values of coverage to those of a buffer with 1% SDS. Increasing the SDS concentration increases coverage values, but for this variant, the median value is slightly higher for samples with a lower amount of material. For kidney stones, applying SDS-based lysis buffers yields very similar coverage values.

In contrast, for the RIPA buffer, these values are significantly lower, especially for samples incubated on ice. In the case of salivary glands, using RIPA buffer yields extremely high values of coverage for some proteins; however, the median value for these variants is generally lower compared to those obtained with SDS-containing buffers. There are no significant differences, but the values are higher for the buffer with 1% SDS. The shape of violin plots presenting coverage distribution for proteins extracted from *R. ornithinolytica* samples is very similar among them, regardless of which variant of lysis buffer was applied. However, the values are generally higher for samples treated with RIPA buffer. Comparing two applied incubation temperatures, the median percentage values of coverage are better for samples treated with RIPA buffer on ice, particularly for those with a higher volume of material (50 µL). In contrast, for heated RIPA, these values are lower, regardless of the volume.

According to statistical analysis, in the case of only four comparisons (saliva - 1% SDS, saliva – 3%, saliva–RIPA (hot), *R. ornithinolytica*–RIPA (cold)), the differences between lower and higher amounts of the tested materials are not statistically significant (*p* > 0.05). Comparing SDS-based buffers and RIPA buffer, in most cases, the differences between these two groups are statistically significant (*p* < 0.05). The results of the proteomic analysis are presented in Supplementary Material S1 (Supplementary Tables S1–S5), and the results of the statistical analysis are shown in Supplementary Materials S6 (Supplementary Tables S1, S2).

### 3.2 Optimisation of the duration of cycles

**FIGURE 3:**
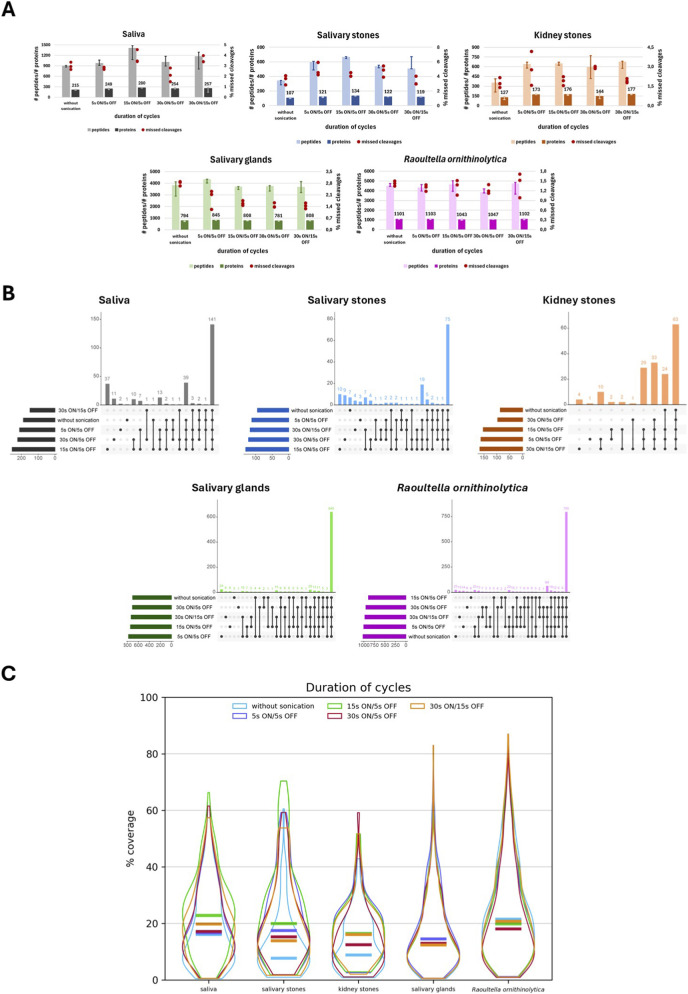
Graphical representation of cycle duration optimisation. **(A)** Bar charts presenting the number (median) of identified peptides and proteins for each applied duration of sonication cycles for different types of biological material: saliva, salivary stones, kidney stones, salivary glands and *Raoultella ornithinolytica*. Red dots present the % value of missed cleavages detected for three technical replicates of each applied variant; **(B)** Upset plots presenting overlapping of identified proteins for each applied for each duration of sonication cycles for different types of biological material: saliva, salivary stones, kidney stones, salivary glands and *Raoultella ornithinolytica*; **(C)** Violin plots presenting the distribution of percentage values of sequence coverage for identified proteins, comparing them to applied protein databases for different types of biological material: saliva, salivary stones, kidney stones, salivary glands, and *Raoultella ornithinolytica*. Horizontal lines show the median value.

To determine the optimal duration of sonication cycles, the number (median) of identified peptides and proteins, as well as the percentage of missed cleavages, is first verified for three technical replicates (Figure 3A).

The results obtained for saliva samples are similar across all applied conditions; however, the best results are associated with a 15s ON/5 s OFF cycle, particularly in terms of the number of detected peptides. Unfortunately, the number of missed cleavages is the highest for this variant. For control samples (without sonication), the lowest number of peptides and proteins was detected. A similar situation was observed for salivary stones; however, in this case, the issue of missed cleavages appears to be better addressed with a 15s ON/5s OFF cycle. Sonication also has a positive influence on the number of identified peptides and proteins in the case of kidney stones. For this type of material, applying 5s ON/5s OFF, 15s ON/5s OFF, and 30s ON/15s OFF cycles enables high-quality protein extraction at nearly the same level. However, applying the 5s ON/5 s OFF cycle may result in a high number of detected missed cleavages. Analysing the results obtained for salivary gland samples, it is evident that sonication may aid in the identification of a higher number of peptides and proteins, with lower values of detected missed cleavages. In the case of a 30s ON/5 s OFF cycle, the extraction efficiency is even worse. For *R. ornithinolytica* samples, all the applied conditions have a similar influence on protein extraction, making it difficult to assess which variant of lysis is the most favourable.

To determine if the choice of various cycles may aid in the extraction and identification of different proteins, upset plots were prepared (Figure 3B). Again, proteins identified as overlapping in three replicates of each variant were chosen. The same 141 proteins were extracted from saliva samples for all applied conditions. Interestingly, a set of 39 proteins was extracted after applying all the variants, even without sonication, but not after applying a 30s ON/15s OFF cycle. A 15s ON/5 s OFF cycle appears to be the most favourable–it allows for the extraction of 37 more proteins. In the case of salivary stone samples, 75 proteins were consistently extracted across all conditions, and 19 proteins were identified explicitly in samples processed with sonication.

There are no significant differences between the samples; however, considering repeatability, the highest quality extraction is associated with applying the 15s ON/5s OFF and 30s ON/5s OFF cycles. By performing protein extraction on kidney stone samples, it was possible to detect 63 proteins that are commonly found in the human body. Lysis without sonication, combined with a 30 s ON/5 s OFF cycle, appears to be the least favourable–firstly, the application of any sonication cycle allows for the detection of 33 specific proteins. Secondly, without the 30s ON/5 s OFF cycle, 24 more proteins were extracted.

Moreover, applying the remaining three cycles, for which the results are quite similar to each other, it is possible to obtain 29 additional proteins. In the case of salivary gland samples, a high number (640) of the same proteins were detected, no matter which variant was applied. Besides that, the results for all of them are quite similar; however, it appears that a 5-s ON/5-s OFF cycle is the most favourable for repeatability of analysis. The results are almost the same for *R. ornithinolytica* samples; however, surprisingly, the best results were obtained when lysis was performed without sonication. Moreover, by choosing any option other than a 15s ON/5 s OFF cycle, it is possible to extract 64 more proteins.

To validate different conditions of the protein extraction process, the percentage values of sequence coverage for overlapped proteins were compared. Figure 3C presents violin plots, which show the distribution of coverage values for different protocol variants and sample types. For saliva samples, analysing the shape of violin plots and median values, it is possible to obtain the best coverage by applying 15 s ON/5 s OFF (*p* < 0.05) and 30 s ON/15 s OFF sonication cycles. The remaining three conditions have a similar influence on the coverage of extracted proteins; however, lysis without sonication is the least favourable. It is also evident in the cases of salivary stones and kidney stones, where standard protein extraction without sonication yields lower coverage values (p-values for almost all comparisons for both samples <0.05). Moreover, for both types of biological material, the application of a 15s ON/5 s OFF cycle yields the best coverage of extracted proteins.

On the other hand, for salivary gland and *R. ornithinolytica* samples, the shapes of the violin plots and median values are very similar across all tested conditions, indicating that the application of different lysis variants does not significantly affect protein coverage. Despite similar values, it is notable that in the case of salivary gland samples, the cycle with the best distribution of coverage values is a 5-s ON/5-s OFF cycle (*p* < 0.05). However, for *R. ornithinolytica* samples, the most favourable variant of lysis is one that does not involve sonication. The results of the proteomic analysis are presented in Supplementary Materials S2 (Supplementary Tables S1–S5). The results of the statistical analysis are shown in Supplementary Materials S6 (Supplementary Table S3).

### 3.3 Optimisation of sonication amplitude

**FIGURE 4:**
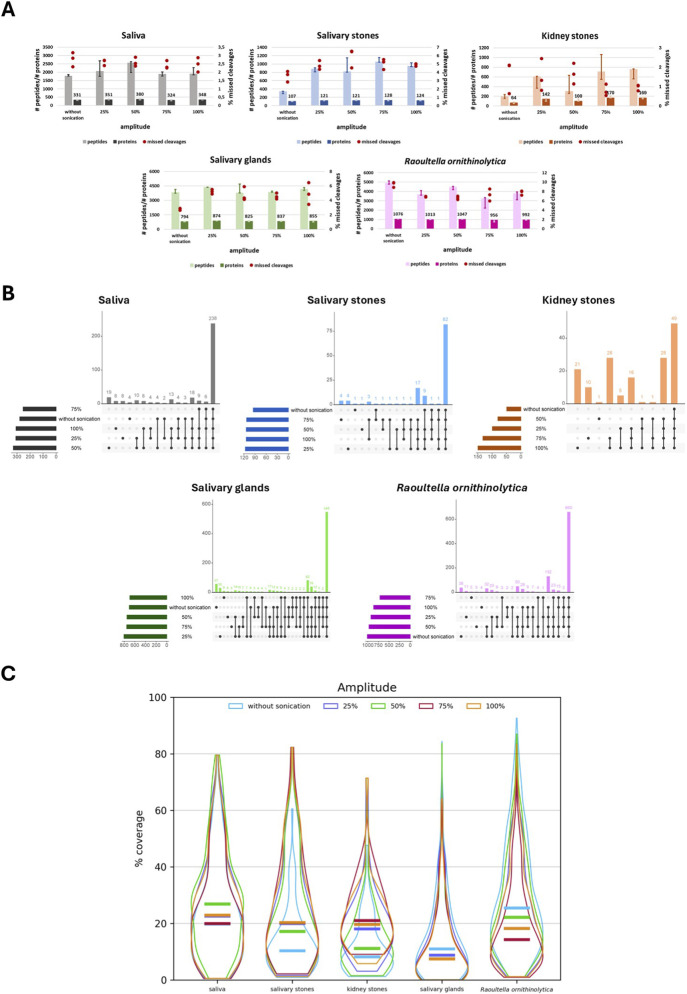
Graphical representation of sonication amplitude optimisation: **(A)** Bar charts presenting the number (median) of identified peptides and proteins for each applied sonication amplitude for different types of biological material: saliva, salivary stones, kidney stones, salivary glands and *Raoultella ornithinolytica*. Red dots present the % value of missed cleavages detected for three technical replicates of each applied variant. **(B)** Upset plots showing the overlap of identified proteins for each applied sonication amplitude across different types of biological material: saliva, salivary stones, kidney stones, salivary glands, and *Raoultella ornithinolytica*. **(C)** Violin plots presenting the distribution of percentage values of sequence coverage for identified proteins, comparing them to applied protein databases for different types of biological material: saliva, salivary stones, kidney stones, salivary glands, and *Raoultella ornithinolytica*. Horizontal lines show the median.

The next stage of the optimisation process is the choice of sonication amplitude value. First, the number of identified peptides and proteins, as well as the percentage value of detected missed cleavages, were checked for each applied variant of the lysis process for different types of samples (Figure 4A).

Analysing the results of peptide and protein extraction from saliva samples, it is evident that the least favourable values were obtained for variants without sonication and with a 75% sonication amplitude. Application of 25% and 100% amplitudes results in lysis with similar efficiency. The most optimal amplitude is 50%, but unfortunately, the level of detected missed cleavages is relatively high for this variant. Extraction of proteins from salivary stones is significantly enhanced by the application of sonication, especially at higher amplitude values, such as 75% and 100%. Unfortunately, the number of detected missed cleavages is higher for these variants of the protocol compared to lysis without sonication.

On the other hand, in the case of kidney stones, the percentage of present missed cleavages is lower for samples processed with stronger sonication (75% and 100%), and the number of extracted peptides and proteins is also higher for these amplitudes. Enhancement of the lysis process for salivary gland samples yields better values in terms of the number of detected peptides and proteins; however, this difference is not statistically significant compared to samples treated in the standard manner. However, the missed cleavages issue appears to be the best option for the process without sonication. The situation is again different for *R. ornithinolytica* samples–application of sonication does not improve extraction. Moreover, higher values of amplitude worsen the efficiency of this process, but the number of missed cleavages is reduced.

The second step in assessing and choosing the most favourable sonication amplitude for the protein extraction procedure involves checking overlapping proteins to develop the possibility of identifying more proteins by applying different intensities of sonication (Figure 4B). In the case of saliva samples, 238 the same proteins were identified for all of the applied lysis variants. Then, the differences between them are not significant, but choosing a 50% amplitude allows for the extraction of 19 more proteins, and this amplitude value appears to be the most favourable. It also appears very similar for salivary stone samples – 82 identified proteins were the same across all protocols; however, it is challenging to differentiate them. However, the application of any sonication amplitude allows for the extraction of 17 more proteins compared to samples processed without sonication. The results obtained for kidney stones are more varied – 49 proteins were common across all amplitude intensity values, and 28 additional proteins were extracted by sonication, regardless of the chosen amplitude. The 75% and 100% amplitudes appear to be the most advantageous, and by applying these conditions, 28 more proteins were identified.

What is more, after sonication at 75% amplitude, 10 extra proteins were extracted, and at 100%, 21 more proteins. For the salivary glands, the same proteins were detected in all of the lysis variants. Applying any intensity of sonication, it was possible to extract 82 more proteins compared to lysis without sonication. However, the set of 39 proteins is unique to unenhanced lysis and lysis enhanced by 25%, 50%, and 100% intense sonication. Among all the tested values, the 25% amplitude appears to be the most favourable – 30 unique proteins were detected for this protocol variant. However, by applying lysis without sonication, an extra set of 57 proteins was obtained. In the case of *R. ornithinolytica* samples, 660 proteins were common to all tested protocols. The least advantageous is a 75% intense amplitude; without this value, it was possible to extract 132 more proteins, regardless of the chosen other protocol. Again, in contrast to other types of biological material, a lysis protocol without sonication is the most favourable, and the application of this variant allows for the detection of 38 unique proteins.

The final step in selecting the optimal sonication amplitude is comparing the percentage coverage values of sequences for overlapping proteins. The results are presented as violin plots (Figure 4C). The shapes of violin plots prepared for differently processed saliva samples are similar to each other; however, the highest median value was obtained using a 50% amplitude, and only for this value are the differences statistically significant. Applying 75% amplitude or processing the samples without sonication results in the lowest sequence coverage. In the case of salivary stone samples, the worst results for sequence coverage are obtained from samples processed without sonication (*p*-values for all comparisons <0.05). For all tested variants of sonication intensity, the values are almost the same, as evident from the shape of the violin plots, the median values, and p-values, especially when applying 25%, 75%, and 100% amplitudes–it is difficult to determine which variant is the best. More noticeable differences between the tested protocols are in the case of kidney stone samples. The lowest values are correlated with skipping sonication or using a 50% amplitude (*p* < 0.05). The remaining variants are quite similar, but the 75% intensity of sonication appears to be the most favourable. The shapes of plots presenting the percentage values of sequence coverage for salivary gland samples processed according to different protocols are almost identical. However, it is noticeable that lysis performed without sonication and with 50% intense sonication yields the highest values. On the other hand, the two most intense sonication amplitudes (75% and 100%) cause the lowest sequence coverage. The same situation applies to *R. ornithinolytica* samples; however, the differences between the tested variants are more significant in this case (*p*-values for almost all comparisons >0.05). The results of the proteomic analysis are presented in Supplementary Materials S3 (Supplementary Tables S1–S5). The results of the statistical analysis are shown in Supplementary Materials S6 (Supplementary Table S4).

### 3.4 Optimisation of sonicator-aided FASP digestion approach

**FIGURE 5:**
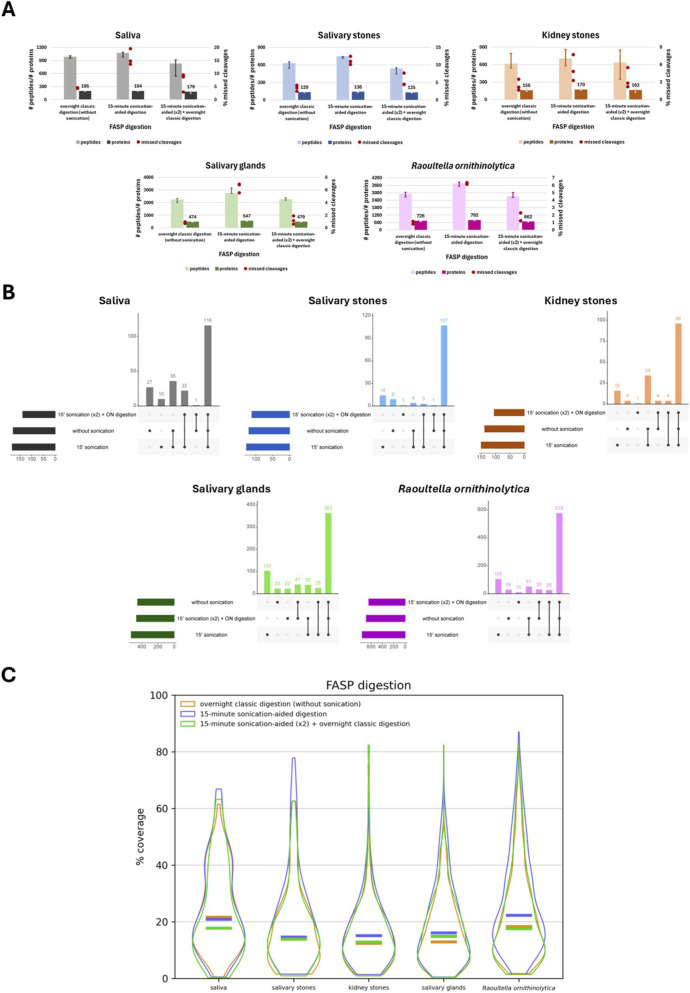
Graphical representation of sonicator-aided FASP digestion approach optimisation. **(A)** Bar charts presenting the number (median) of identified peptides and proteins for each applied FASP digestion variant for different types of biological material: saliva, salivary stones, kidney stones, salivary glands and *Raoultella ornithinolytica*. Red dots present the % value of missed cleavages detected for three technical replicates of each applied variant. **(B)** Upset plots presenting the overlapping of identified proteins for each applied FASP digestion variant for different types of biological material: saliva, salivary stones, kidney stones, salivary glands and *Raoultella ornithinolytica*. **(C)** Violin plots presenting the distribution of percentage values of sequence coverage for identified proteins, comparing them to applied protein databases for different types of biological material: saliva, salivary stones, kidney stones, salivary glands, and *Raoultella ornithinolytica*. Horizontal lines show median values.

After optimising the lysis conditions, the next step was to choose the best digestion approach. Three various protocols were compared: standard overnight FASP digestion (without sonication), 15-min sonication-aided FASP digestion and overnight FASP digestion combined with two 15-min sonication cycles (before and after overnight digestion). First, the number of identified peptides and proteins was compared between the tested protocols (Figure 5A).

Starting from saliva samples, the application of standard overnight digestion and 15-min sonication-aided digestion results in a similar number of detected proteins. However, the number of missed cleavages is higher after short-time digestion with sonication, but more peptides were identified in this way. The combination of classic overnight sample processing with 15-min sonication cycles does not have a positive influence on the number of detected proteins; the percentage value of missed cleavages is also higher compared to classic digestion. In the case of salivary stones, the combined protocol is also less efficient. The 15-min digestion with sonication appears to be the most favourable–the number of identified peptides is slightly higher than after basic protocol application, but for this variant, more than twice as many missed cleavages were detected.

On the other hand, for kidney stone samples, the least favourable protocol was standard overnight digestion without sonication. However, the differences between the tested approaches were not significant, also in the case of detected missed cleavages. More visible differences are present in the case of salivary gland and *R. ornithinolytica* samples–for both types of biological material, the best results were obtained after application of short-time sonication-aided digestion. However, the number of found missed cleavages is approximately six times higher for both types of sample compared to other protocols. Analysing the remaining treatment variants, the results were similar in the case of salivary glands; however, for bacterial samples, the application of standard overnight digestion had a positive impact on the number of identified proteins.

To check if it is possible to identify other proteins by applying various digestion protocols, the upset plots were prepared (Figure 5B). For saliva samples, the same proteins were detected in all three tested approaches. Choosing the option without sonication or with short-time sonication, 36 more proteins were detected for both variants: 27 for the first variant and 10 for the second. 107, the application of all protocols identified the same proteins. Then, the differences between them were not significant, but the 15-min sonication-aided digestion appeared to be the most effective. This protocol appears to be the most favourable, also in the case of kidney stone samples, as 16 additional proteins were detected using this option.

Additionally, 34 more proteins were identified for this type of digestion and treatment without sonication enhancement, where 96 of the same proteins were present in all tested protocols. Digestion of the salivary gland samples, employing three different approaches, enables the identification of 363 common proteins. Here, the differences between variants are more pronounced, but the most advantageous approach appears to be 15-min digestion with sonication–by choosing only this protocol, it is possible to identify 103 additional proteins. The situation is also similar for *R. ornithinolytica* samples–the standard set of detected proteins contains 576 of them, but applying short-time sonication-aided sample processing reveals 105 more proteins.

The final step in assessing the tested digestion protocols was to compare the percentage values for the coverage of identified protein sequences (Figure 5C). Analysing the proteins detected in saliva samples, the best values of coverage were obtained by applying the classic overnight digestion without sonication, slightly less optimal values were present in the case of 15-min sonication-aided samples processing. The shapes of violin plots generated for salivary stone, kidney stone, and salivary gland samples had a quite similar pattern–the most favourable coverage values were generated for samples processed according to the protocol with short digestion enhanced by sonication. The least favourable was standard digestion without sonication. In the case of *R. ornithinolytica* samples, the 15-min digestion was also the best option; however, the least favourable protocol was a combination of overnight digestion and sonication. The statistically significant differences were noticeable only in the case of salivary glands and *R. ornithinolytica* samples. The results of the proteomic analysis are presented in Supplementary Materials S4 (Supplementary Tables S1–S5). The results of the statistical analysis are shown in Supplementary Materials S6 (Supplementary Table S5).

### 3.5 Comparison of sonicator-aided lysis and barocycler-aided lysis

**FIGURE 6:**
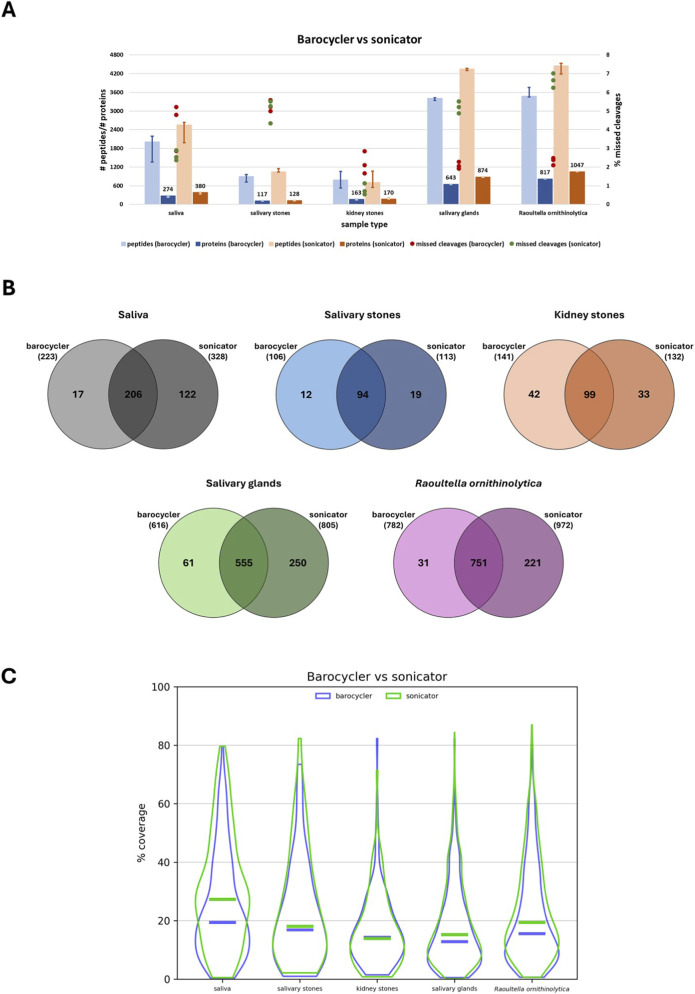
Graphic comparison of sonicator-aided lysis and barocycler-aided lysis. **(A)** Bar chart presenting the number (median) of identified peptides and proteins depending on the applied type of enhancing the sample lysis for different types of biological material: saliva, salivary stones, kidney stones, salivary glands and *Raoultella ornithinolytica*. Red dots present the % value of missed cleavages detected for three technical replicates of each applied variant. **(B)** Venn diagrams illustrating the overlap of identified proteins depending on the type of sample lysis enhancement for various biological materials: saliva, salivary stones, kidney stones, salivary glands, and *Raoultella ornithinolytica*. **(C)** Violin plots presenting the distribution of percentage values of sequence coverage for identified proteins, comparing them to applied protein databases for different types of biological material: saliva, salivary stones, kidney stones, salivary glands, and *Raoultella ornithinolytica*. Horizontal lines show median values.

To verify the validity of using a sonicator to enhance the efficiency of the lysis process, two protocols based on sonication and pressure cycling technology (PCT) were compared (see Matherials and methods, 2.2 Protein extraction). To compare these approaches, the samples were treated as similarly as possible–most importantly, the same lysis buffers were used for each type of sample, as established earlier. First, the number of detected peptides, proteins, and missed cleavages was compared between these two approaches (Figure 6A).

After processing the saliva sample, it is evident that using a sonicator for lysis enhancement allows for the detection of a higher number of peptides and proteins; for the latter, the difference is over 100 (274 vs. 380). Besides, the number of found missed cleavages is also more favourable by applying sonication instead of high pressure. In the case of both types of stone samples, the difference is not significant, but sonicator-assisted lysis remains more advantageous. However, for kidney stones, the number of detected peptides was higher after processing the samples with the barocycler. Regarding the missed cleavages, their detection level was almost the same for saliva stone samples; however, for kidney stones, it improved after sonication was applied for protein extraction. Processing the salivary gland and *Raoultella ornithinolytica* samples according to the protocol based on sonication is preferable; in the case of both types of samples, the difference exceeds 200 proteins. Unfortunately, the percentage of detected missed cleavages is significantly higher for this option.

To assess whether the use of different enhancement strategies influences the number of detected proteins, protein sets obtained using sonicator-aided lysis were compared with those obtained using barocycler-aided lysis. For this purpose, Venn diagrams were generated based on proteins consistently identified in three technical replicates of each approach (Figure 6B). For saliva samples, 206 detected proteins were the same for both tested protocols; however, more unique proteins were identified after sonication (122 vs. 17). In the case of salivary stones, besides 94 common proteins, which make up most of them, the number of specific proteins for the used approach proteins is not significantly different, but more advantageous looks sonication. I*n* contrast, protein sets obtained from kidney stone samples differed more markedly between the two lysis approaches. A total of 99 proteins were consistently identified using both methods, whereas 42 proteins were uniquely detected following sonicator-aided lysis and 33 proteins were identified exclusively after barocycler-aided lysis.

For salivary gland and *R. ornithinolytica* samples, the majority of detected proteins overlapped between the two approaches; however, method-specific protein sets were also observed. In salivary gland samples, 555 proteins were consistently identified following sonicator-aided lysis, while in *R. ornithinolytica* samples, 751 proteins were consistently detected after barocycler-aided lysis. These differences likely reflect distinct mechanisms of protein release promoted by mechanical disruption during sonication *versus* pressure-induced cell and matrix permeabilisation during pressure cycling, which may preferentially expose different subsets of proteins depending on the structural properties of the analysed material. Additionally, for both sample types, the application of sonication is more advantageous–sample processing with a barocycler allows for the extraction of fewer proteins.

Lastly, the percentage values of sequence coverage for extracted proteins using sonication and high-pressure cycling as enhancement methods were compared (Figure 6C). The most significant difference is visible in violin plots presenting the coverage values of saliva proteins–the application of sonication is more advantageous for enhancing lysis (*p* = 9.90E-05). In the case of salivary stone samples, sonication-aided lysis is also preferable; however, the difference is not significant (*p* = 0.27). On the other hand, the coverage values of proteins from kidney stones are slightly higher after the application of PCT (*p* = 0.64). For the last two types of samples tested–salivary glands (*p* = 0.002) and *R. ornithinolytica* (*p* = 0.00014) – the choice of sonication-aided protein extraction appears better in terms of the obtained percentage coverage values. The results of the proteomic analysis are presented in Supplementary Materials S5 (Supplementary Table S1). The results of the statistical analysis are shown in Supplementary Materials S6 (Supplementary Table S6).

## 4 Discussion

**FIGURE 7:**
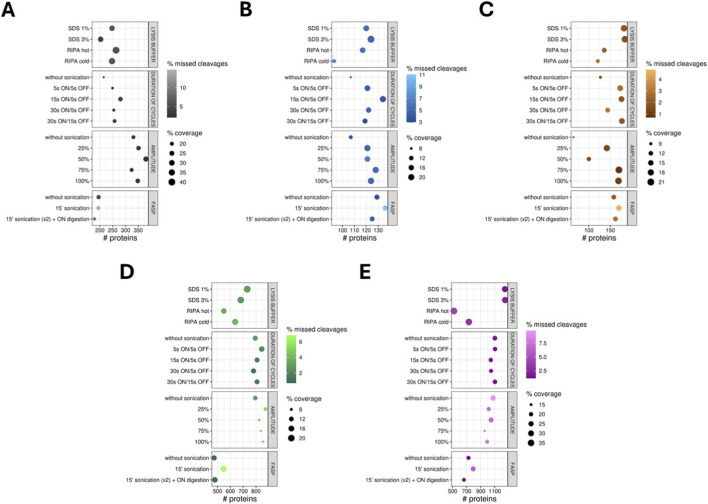
Bubble plots presenting the summary of results from the optimisation of a sample processing protocol using a sonicator. The optimisation was performed by processing different types of biological material: saliva **(A)**, salivary stones **(B)**, kidney stones **(C)**, salivary glands **(D)** and *Raoultella ornithinolytica* **(E)**. The x-axis shows the number of identified proteins. The size of bubbles is associated with the median values of sequence coverage for identified proteins, compared to the proteins in the applied protein databases. The intensity of bubble fill colour is associated with the median percentage value of missed cleavages detected for three technical replicates of each applied variant.

Proteomic analysis of pathological calcifications, such as those found in salivary and kidney stones, is challenging due to the complex composition of these biomaterials and the difficulty in obtaining reproducible protein yields. Nevertheless, understanding their proteomic signatures is of high clinical importance, as it may provide insights into disease mechanisms and support the discovery of biomarkers for stone-related disorders. An optimised protocol for protein extraction and digestion is crucial for sample preparation and obtaining satisfactory results. Saliva and salivary stones are relatively uncommon sample types, and few validated protocols are available for their processing. Because these samples are demanding and challenging, our goal was to develop, optimise, and validate a processing protocol to achieve the most efficient result possible. Therefore, we decided to use a sonicator for sample processing, hoping this enhancement method might help identify more proteins. Our study compared different lysis buffers, sample amounts, and sonication cycles, demonstrating that optimisation of these parameters substantially influences peptide and protein identification, missed cleavage rates, and sequence coverage. Such improvements are not only technical but also translational, as more reliable proteomic data enhance the likelihood of identifying clinically relevant biomarkers. Additionally, Figure 7 summarises the entire optimisation procedure.

### 4.1 Optimisation of the amount of biological material and lysis buffer

Optimisation of the amount of biological material is particularly important in studies based on clinical samples, where sample availability is often limited. The observed improvement in proteomic performance with increasing sample input indicates that higher material loads facilitate more efficient protein extraction and digestion. However, the relatively small differences between the tested amounts suggest that acceptable proteomic coverage can still be achieved when working with reduced sample quantities, which is relevant for clinical scenarios where material is scarce.

The choice of lysis buffer strongly influenced extraction efficiency and was highly dependent on the type of biological material analysed. Differences observed between saliva, stone-derived samples and glandular tissue likely reflect their distinct biochemical composition and structural organisation. Strongly denaturing, SDS-containing buffers appeared more suitable for mineralised or dense matrices, where disruption of protein–matrix interactions is required. In contrast, saliva benefited from the use of heated RIPA buffer, which may enhance solubilisation of soluble and membrane-associated proteins. For bacterial samples, lower detergent concentrations were sufficient, indicating that excessive denaturation is not required for efficient protein recovery from this material type.

### 4.2 Choice of duration of cycles

Variation in sonication cycle duration revealed that optimal mechanical disruption is sample dependent. Differences between saliva, stone material and tissue samples likely arise from their heterogeneous physical properties, including hardness, cellular density and mineral content. Shorter sonication cycles may be sufficient to disrupt softer or more soluble matrices, whereas denser and mineralised samples may require prolonged mechanical treatment to improve protein accessibility.

The lack of clear improvement for bacterial samples following sonication suggests that mechanical disruption may not be a limiting factor for protein extraction in this case, and that standard chemical lysis is adequate. These findings highlight the importance of tailoring sonication parameters to the specific structural characteristics of the analysed material rather than applying a uniform approach.

### 4.3 Choice of sonication amplitude

Similar to sonication duration, the effect of sonication amplitude varied across sample types, confirming that no universal amplitude setting is optimal for all biological materials. Higher amplitudes may enhance protein release from rigid or calcified structures but may also increase the risk of protein degradation or incomplete digestion. Conversely, lower amplitudes may preserve protein integrity but provide insufficient disruption for dense matrices.

The limited benefit of sonication for *R. ornithinolytica* further supports the conclusion that bacterial samples do not require intensive mechanical treatment during protein extraction. Overall, these observations reinforce the need for balanced optimisation that maximises protein accessibility while minimising adverse effects on downstream proteolysis.

### 4.4 Choice of FASP digestion approach

Evaluation of different FASP-based digestion strategies demonstrated that accelerated, sonication-assisted digestion can improve processing efficiency without substantial loss of proteomic depth for certain sample types. However, the observed increase in missed cleavages following shortened digestion times indicates that faster protocols may compromise proteolytic completeness, as previously reported for rapid digestion approaches.

For more complex and mineralised samples, extended digestion combined with intermittent sonication appeared to improve overall performance, suggesting that prolonged enzyme access is necessary to achieve efficient cleavage. These observations are consistent with earlier studies highlighting the trade-off between digestion speed and proteolytic efficiency in proteomic workflows (Carvalho et al., 2020; Wiśniewski et al., 2009a; Wiśniewski, 2019; Wiśniewski et al., 2009b).

### 4.5 Missed cleavages

Minimisation of missed cleavages is a critical quality parameter in proteomic workflows, as incomplete proteolysis directly affects sequence coverage, identification confidence and quantitative accuracy (Hunter, 2007; Szklarczyk et al., 2023; “Created with BioRender; Siepen et al., 2007; Sun et al., 2021; Thiede et al., 2000; McGurk et al., 2020; Kristensen et al., 2022). Increased numbers of missed cleavages reduce peptide detectability, introduce ambiguity in protein inference and negatively impact quantitative analyses.

The elevated occurrence of missed cleavages observed for samples processed with RIPA buffer likely reflects the presence of detergents that interfere with protease activity, emphasising the trade-off between extraction efficiency and digestion quality. In contrast, SDS-containing buffers provided a more favourable balance between efficient protein solubilisation and proteolytic performance, particularly for complex and mineralised materials. The variable influence of sonication parameters on missed cleavage rates further suggests that excessive mechanical treatment may negatively affect enzymatic digestion, underscoring the need for careful optimisation of sample preparation workflows in clinically oriented proteomic studies (Siepen et al., 2007; Sun et al., 2021; Thiede et al., 2000; McGurk et al., 2020; Kristensen et al., 2022; McDonnell et al., 2022; Deng et al., 2018; Fierro-Monti et al., 2024).

Enhancing the digestion process with sonication increases the percentage of detected missed cleavages, most notably in the 15-min digestion protocol, where the difference is significant compared to the other two protocols. When comparing classic digestion with an approach that combines standard protocol and sonication, there are no significant differences in the number of detected missed cleavages; however, the protocol without sonication is more advantageous. It suggests that even if the 15-min sonication-aided digestion improves the number of detected peptides and proteins, the high amount of present missed cleavages and attempts to correct them using proteomic software may lead to an increased number of false positives, consequently detecting proteins that are not present.

Comparing the number of detected missed cleavages after sonicator-aided lysis and barocycler-aided lysis reveals no clear pattern, with varied results. For saliva and kidney stone samples, sonication appears to be a more optimal choice. The situation differs for salivary gland and *R. ornithinolytica* samples, where the values indicating the level of detected missed cleavage sites are lower. For salivary stones, the level of detected missed cleavage sites is the same for both tested approaches.

### 4.6 Comparison of sonicator-aided lysis and barocycler-aided lysis

To verify the validity of sonication-aided lysis, the results were compared with those obtained from samples processed using a barocycler, a standard method employed to enhance sample processing efficiency (Pirog et al., 2021; Olszowy et al., 2013; Lucas et al., 2019). Sonication allowed for the extraction of a higher number of peptides and proteins from all tested biological materials. Regarding the detected missed cleavages, it is challenging to definitively state which protocol is more advantageous. Since sonication enabled the identification of more proteins, and the majority of overlapping proteins are consistent between both protocols, processing samples using both approaches to increase the total number of detected proteins does not appear necessary, though it may enhance the results. Additionally, in terms of protein coverage, the sonicator appears to be a more reasonable choice; however, for kidney stones, the percentage values of sequence coverage are more optimal after sample processing using pressure cycling technology.

### 4.7 Comparison of proteins detected in saliva and salivary stone samples

**FIGURE 8:**
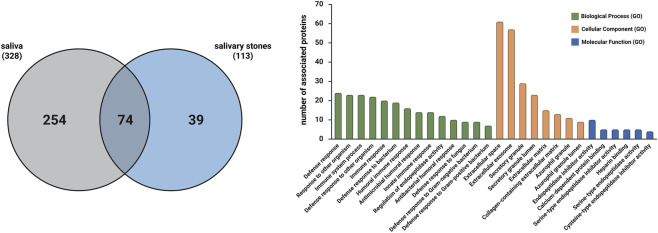
Venn diagram illustrating the overlap of proteins identified in saliva and salivary stones, and bar plot presenting the results of enrichment analysis for the overlapped proteins detected in saliva and salivary stones, considering Biological Process GO (BP), Cellular Component GO (CC) and Molecular Function GO (MF) terms.

Finally, we aimed to assess the validity of comparing proteins identified in two different clinical materials associated with sialolithiasis: saliva and salivary stones. The Venn diagram (Figure 8) illustrates this comparison, showing 74 proteins that overlap.

Analysing the enrichment analysis, most of the detected terms are consistent with our previous studies (Czaplewska et al., 2021; Musiał et al., 2023). The results are presented in Figure 8, where bars show the number of proteins associated with each Gene Ontology enrichment term. Focusing first on Biological Process GO terms, some of the common proteins are associated with the immune response to bacterial presence, suggesting that bacteria may influence homeostasis in the salivary gland area, leading to stone formation. Additionally, the Regulation of endopeptidase activity term was identified in a group of 12 proteins, suggesting potential disturbance in lipid levels (Muangman et al., 2003). Cellular Component GO enrichment indicates that almost all proteins are associated with extracellular compartments, which again suggests that sialolith formation may result from the presence of neutrophil extracellular traps. However, the *Neutrophil Extracellular Trap Formation (NETs)* KEGG pathway was not detected this time. According to the Molecular Function GO database, some proteins are associated with endopeptidase activity, which supports the theory that changes in lipid levels contribute to the biocalcification process. Furthermore, some Reactome pathways were also identified during the enrichment analysis, further supporting the previously described potential reasons for sialolithiasis. Analysing the Reactome database, the following pathways were identified: *Immune System* (27 proteins), *Innate Immune System* (25 proteins), *Neutrophil Degranulation* (23 proteins), and *Antimicrobial Peptides* (11 proteins).

**FIGURE 9:**
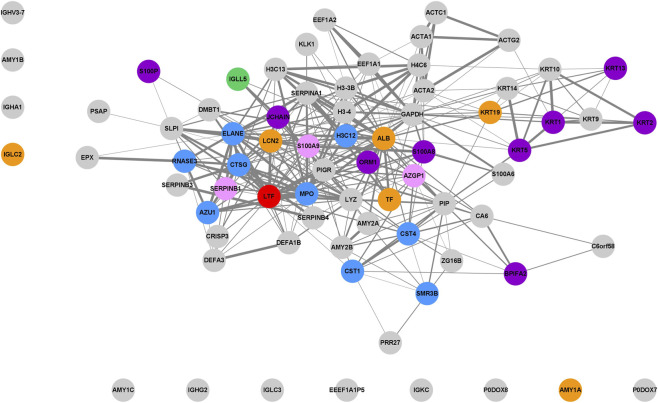
The Cytoscape visualisation of the STRING-generated network is composed of experimentally verified protein–protein interactions among the overlapped proteins detected in saliva and salivary stones. Fill colours correspond with overlapping of proteins identified in different types of salivary stones: blue–CAL + LIP + MIX, green–CAL + LIP, orange–CAL + MIX, pink–LIP + MIX, purple–only CAL, red–only LIP.

Overlapped proteins identified in saliva and salivary stones were visualised as a STRING-generated network of their interactions in Figure 9. This set of proteins was compared to proteins detected in sialoliths during our last study (Musiał et al., 2023), considering their classification into one of three groups: CAL, LIP, and MIX. There are nine proteins, marked in blue, which were detected in all types of salivary stones, making them the most crucial: Cystatin-S (CST4), Submaxillary gland androgen-regulated protein 3B (SMR3B), Cystatin-SN (CST1), Myeloperoxidase (MPO), Cathepsin G (CTSG), Histone H3.1 (H3C12), Eosinophil cationic protein (RNASE3), Neutrophil elastase (ELANE), Azurocidin (AZU1). Cystatin-S (CST4) and Cystatin-SN (CST1), common proteins in saliva, inhibit human cathepsins and bind calcium, altering homeostasis (Baron et al., 1999). Cathepsin G (CSTG), a neutrophil serine protease, possesses antimicrobial activity (Alford et al., 1990; Bangalore et al., 1990; Kavanaugh et al., 2021), is present in NETs (Thomas et al., 2014), and may modulate calcium ion levels (Garofalo and Peterson, 1989) and lipid metabolism (Houben et al., 2017). Myeloperoxidase (MPO), with activity in defending against bacteria (Davies, 2011; Klebanoff, 2005), can also be found in NETs (Lubahn et al., 2014). Furthermore, this protein has a high affinity for calcium (Shin et al., 2001) and may influence lipid peroxidation (Zhang et al., 2002). Eosinophil cationic protein (RNASE3) has cytotoxic activity against pathogens (Torrent et al., 2008). One of the proteins, Submaxillary gland androgen-regulated protein 3B (SMR3B), inhibits endopeptidase activity, which may influence lipid levels (SMR3B submaxillary gland androgen regulated). Azurocidin (AZU1), which regulates macrophage function during immune response, is calcium-dependent (Soehnlein and Lindbom, 2009). Both Histone H3.1 (H3C12) and Neutrophil elastase (ELANE) are associated with NETs; the former influences chromatin decondensation (Hirose et al., 2014), while neutrophils secrete the latter during inflammation (de Mello Mazetto et al., 2022).

There is one protein (green), Immunoglobulin lambda-like polypeptide 5 (IGLL5), which is common to both CAL and LIP stones. Six proteins (orange) were found in both CAL and MIX sialoliths: Albumin (ALB), Neutrophil gelatinase-associated lipocalin (LCN2), Keratin, type I cytoskeletal 19 (KRT19), Serotransferrin (TF), Immunoglobulin lambda constant 2 (IGLC2), Alpha-amylase 1A (AMY1A). Protein S100-A9 (S100A9), Zinc-alpha-2-glycoprotein (AZGP1), and Leukocyte elastase inhibitor (SERPINB1) were identified in both LIP and MIX salivary stones. Analysing proteins found only in one type of sialolith, nine were identified in CAL stones (purple Keratin, type I cytoskeletal 13 (KRT13), Keratin, type II cytoskeletal 5 (KRT5), Protein S100-A8 (S100A8), BPI fold-containing family A member 2 (BPIFA2), Keratin, type II cytoskeletal 1 (KRT1), Immunoglobulin J chain (JCHAIN), Keratin, type II cytoskeletal two epidermal (KRT2), Protein S100-P (S100P), Alpha-1-acid glycoprotein 1 (ORM1). Lactotransferrin (LTF). Lactotransferrin (LTF) was present in LIP stones; no protein was unique to MIX sialoliths.

### 4.8 Comparison of proteins detected in salivary stone and kidney stone samples

**FIGURE 10:**
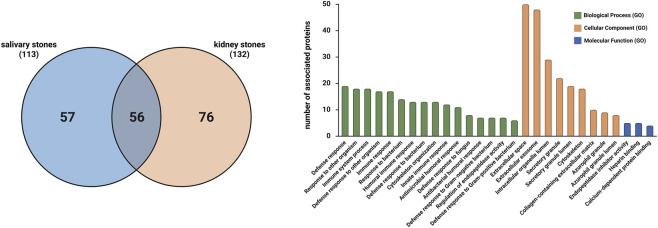
Venn diagram illustrating the overlap of proteins identified in salivary stones and kidney stones, and bar plot presenting the results of enrichment analysis for the overlapped proteins detected in salivary stones and kidney stones, considering Biological Process GO (BP), Cellular Component GO (CC) and Molecular Function GO (MF) terms.

Finally, we examined common proteins in both salivary and kidney stones to identify crucial proteins responsible for the biocalcification process, regardless of the pathological state. The Venn diagram (Figure 10) presents 56 overlapping proteins.

Enrichment analysis performed on this set of proteins yielded results similar to previous analyses (Figure 10). According to the Biological Process GO database, these overlapped proteins are mainly associated with antimicrobial activity and immune response, suggesting that the presence of bacteria and the subsequent immune response may contribute to stone formation. The term *Regulation of endopeptidase activity* was again detected for 7 proteins, indicating that alterations in lipid levels may be responsible for biocalcification, not only in salivary glands but also in other body areas. Regarding the Cellular Component GO database, most proteins are linked to the regulation of extracellular domains, leading us to hypothesise that neutrophil extracellular traps may also play a significant role in the formation of other types of stones in the human body, underscoring the broader clinical significance of these mechanisms. For the Molecular Function GO database, the terms *Endopeptidase inhibitor activity* (5 proteins) and *Calcium-dependent protein binding* (4 proteins) were detected, indicating the potential for disturbances in lipid and calcium metabolism, respectively. Additionally, detected Reactome pathways, such as *Immune System* (25 proteins), *Neutrophil degranulation* (18 proteins), and *Antimicrobial peptides* (9 proteins), further support the hypothesis about the role of bacteria.

**FIGURE 11:**
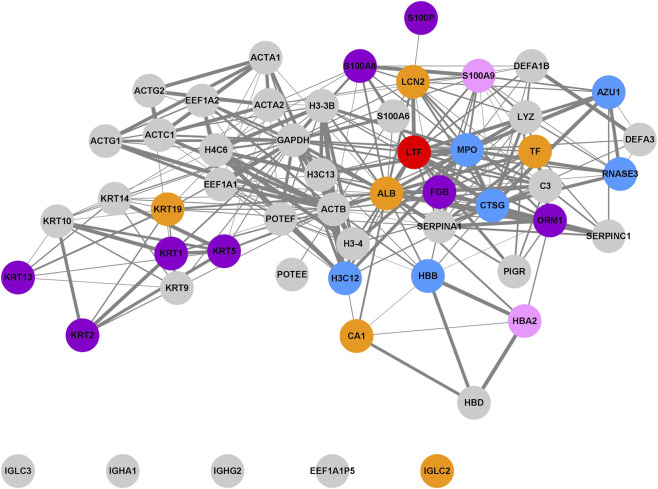
The Cytoscape visualisation of the STRING-generated network is composed of experimentally verified protein–protein interactions among the overlapped proteins detected in salivary stones and kidney stones. Fill colours correspond to the overlapping of proteins identified in different types of salivary stones: blue–CAL + LIP + MIX, orange–CAL + MIX, pink–LIP + MIX, purple–only CAL, and red–only LIP.

Again, the STRING-generated network of interactions between overlapped proteins identified in salivary and kidney stones was prepared (Figure 11). This set of proteins was also compared to sets established earlier for CAL, LIP, and MIX sialoliths. There is a group of six proteins that are common to all three stone types (marked in blue): Haemoglobin subunit beta (HBB), Eosinophil cationic protein (RNASE3), Cathepsin G (CTSG), Histone H3.1 (H3C12), Azurocidin (AZU1), and Myeloperoxidase (MPO). Most of these are the same proteins identified in the comparison between saliva and salivary stones. Six additional proteins were found in both CAL and MIX stones: Albumin, Keratin, type I cytoskeletal 19, Neutrophil gelatinase-associated lipocalin, Carbonic anhydrase 1, Serotransferrin, and Immunoglobulin lambda constant 2. Protein S100-A9 and Haemoglobin subunit alpha were detected in LIP and MIX sialoliths.

Eight proteins were identified as unique to CAL salivary stones: Keratin, type I cytoskeletal 13, Protein S100-A8, Keratin, type II cytoskeletal 5, Keratin, type II cytoskeletal 2 epidermal, Keratin, type II cytoskeletal 1, Fibrinogen beta chain, Protein S100-P, and Alpha-1-acid glycoprotein 1. Only one protein, Lactotransferrin (LTF), was unique for LIP stones.

Ultimately, we wanted to verify if our detected proteins overlapped with some known biomarkers associated with kidney stones. We identified 10 proteins that were previously described as potentially crucial during biocalcification, leading to the formation of kidney stones (Yang et al., 2021). These proteins are: Haemoglobin subunit beta, Eosinophil cationic protein, Cathepsin G, Myeloperoxidase, Protein S100-A8, Albumin, Protein S100-A9, Lactotransferrin, Lysozyme C, and Complement C3. Most of them were mentioned earlier. S100-A8 and S100-A9 are proteins associated with immune response in the presence of bacteria, binding calcium and forming calprotectin (Lehmann et al., 2015; Stříž and Trebichavský, 2004; Avila et al., 2013; Clark et al., 2016). Complement C3 (C3) is also part of the immune system, but additionally plays a role in lipid metabolism (Barbu et al., 2015). Lactotransferrin (LTF), with antimicrobial activity, is dependent on the level of calcium ions (Arnold et al., 1982). Together with Lysozyme (LYZ), an antimicrobial protein, they are part of neutrophil extracellular traps (Lubahn et al., 2014; Manchenko, 2002).

Previous studies have reported proteomic characterisation of stones; however, protocols varied considerably and often resulted in limited reproducibility. By systematically evaluating sample preparation strategies, our work provides a methodological framework that can be applied in future clinical proteomics studies of biocalcifications.

Importantly, our pilot data indicate that optimised workflows can be applied to diverse sample types, from saliva to stone material, enabling integrative analyses across tissues and fluids. It opens possibilities for identifying shared molecular signatures of calcification, which could serve as candidate biomarkers for diagnosis or monitoring.

Epidemiological evidence suggests that salivary stone disease and kidney stone disease may co-occur more frequently than expected by chance (Wu et al., 2016). Population-based case–control and cohort studies have reported an association between sialolithiasis and nephrolithiasis/urolithiasis, supporting the concept of shared systemic or local predispositions to pathological calcification (Jonas et al., 2025). In addition, analyses of stone disease comorbidities and familial aggregation have suggested modest but measurable links between salivary and urinary stone formation (Hemminki et al., 2018). These observations provide a clinical and translational rationale for exploring whether salivary and kidney stones share overlapping molecular components, and whether common biological pathways—such as inflammation-related processes or mineral-binding protein networks—may contribute to calcification across different anatomical sites. At the same time, the strength of this association varies between studies and populations, and current evidence does not support a direct causal relationship, highlighting the need for cautious interpretation of shared molecular features.

### 4.9 Limitation of the studies

The main limitation of this study is the relatively small number of samples, which does not allow for statistical validation of biomarker candidates. Therefore, our findings should be interpreted as proof-of-concept, providing a basis for larger-scale studies rather than definitive clinical conclusions.

Taken together, our results demonstrate that methodological optimisation of proteomic workflows is essential for reliable characterisation of challenging biomaterials. These optimised protocols support future translational studies and may ultimately contribute to the identification of biomarkers and the improvement of clinical management in stone disease and related calcification disorders.

Another limitation of this study is the use of saliva collected from healthy volunteers rather than from patients with salivary stones. Previous studies have shown that the salivary proteome may differ between affected individuals and healthy controls, which could influence direct biological comparisons. In the present work, saliva was included primarily as a complementary and technically challenging biological matrix to support methodological optimisation, rather than as a source for clinical biomarker validation.

Salivary stone samples were collected over an extended period of time, and saliva collection was not part of the initial study design. Consequently, saliva samples from the same patients were not available for analysis. This limitation should be taken into account when interpreting the comparative analyses presented here. Importantly, saliva sampling from patients with salivary stones prior to surgical intervention has been incorporated into the design of ongoing and future studies, which will allow direct and clinically relevant comparisons.

Another limitation of this study is related to the source of salivary gland tissue used for methodological evaluation. Although the majority of salivary stones originate in the submandibular gland, the glandular tissue analysed in this work was derived exclusively from parotidectomy specimens. This choice was driven by practical considerations, as access to submandibular gland tissue suitable for research purposes is limited.

Importantly, salivary gland tissue was not included to model the site of stone formation, but rather to represent a structurally complex human tissue and to assess the applicability of the optimised proteomic workflows across different biological matrices. Nevertheless, differences between parotid and submandibular glands should be taken into account when interpreting these results.

## 5 Conclusion

This pilot study demonstrates that optimised sample preparation workflows enable robust proteomic profiling of challenging biomaterials such as saliva, salivary stones, and kidney stones. By enhancing protein identification and sequence coverage, these protocols establish a methodological foundation for future translational studies. Although limited in sample size, our findings highlight the potential of optimised proteomics to support biomarker discovery and to advance the understanding of pathological calcification in clinical contexts.

By optimising protein extraction and digestion using sonication, we have demonstrated that this technique can enhance sample processing efficiency for certain biological materials. However, it is not feasible to establish a single, universal protocol applicable to all types of biological material; instead, it is crucial to develop and optimise tailored protocols specific to each biological matrix. Notably, in the case of bacterial samples such as *R. ornithinolytica*, sonication did not improve protein extraction efficiency, and conventional processing methods proved more effective. Nevertheless, this observation should not be generalised to all bacterial species, as the effectiveness of sonication may vary significantly across different organisms.

This pilot study demonstrates that optimised sample preparation workflows enable robust and reproducible proteomic profiling of challenging biomaterials, including saliva, salivary stones and kidney stones. By systematically optimising protein extraction and digestion conditions, we improved protein identification efficiency and sequence coverage across diverse sample types.

Although the limited number of samples precludes definitive biomarker validation, the protocols established in this study provide a strong methodological framework for future translational proteomics investigations. These workflows may facilitate integrative analyses of biological fluids and calcified tissues and support subsequent studies aimed at identifying and validating molecular markers associated with pathological calcification and stone disease.

## Data Availability

The data presented in the study are deposited in the ProteomeXchange Consortium via the PRIDE partner repository, accession number PXD069065.
